# Anti-apoptotic Bcl-X_L_ but not Mcl-1 contributes to protection against virus-induced apoptosis

**DOI:** 10.1038/cddis.2016.242

**Published:** 2016-08-18

**Authors:** Michaela Ohmer, Arnim Weber, Gerd Sutter, Katrin Ehrhardt, Albert Zimmermann, Georg Häcker

**Affiliations:** 1 Institute of Medical Microbiology and Hygiene, University Medical Center Freiburg, Freiburg, Germany; 2 Faculty of Biology, University of Freiburg, Freiburg, Germany; 3 Institute for Infectious Diseases and Zoonoses, Ludwig-Maximilians-University Munich, Munich, Germany; 4 Institute of Virology, University Medical Center Freiburg, Freiburg, Germany; 5 Institute for Virology, Medical Faculty, Heinrich-Heine-University Düsseldorf, Düsseldorf, Germany

## Abstract

Infection of mammalian cells with viruses often induces apoptosis. How the recognition of viruses leads to apoptosis of the infected cell and which host cell factors regulate this cell death is incompletely understood. In this study, we focussed on two major anti-apoptotic proteins of the host cell, whose abundance and activity are important for cell survival, the Bcl-2-like proteins Mcl-1 and Bcl-X_L_. During infection of epithelial cells and fibroblasts with modified vaccinia virus Ankara (MVA), Mcl-1 protein levels dropped but the MVA Bcl-2-like protein F1L could replace Mcl-1 functionally; a similar activity was found in vaccinia virus (VACV)-infected cells. During infection with murine cytomegalovirus (MCMV), Mcl-1-levels were not reduced but a viral Mcl-1-like activity was also generated. Infection of mouse macrophages with any of these viruses, on the other hand, induced apoptosis. Virus-induced macrophage apoptosis was unaltered in the absence of Mcl-1. However, apoptosis was substantially increased in infected Bcl-X_L_-deficient macrophages or macrophages treated with the Bcl-2/Bcl-X_L_-inhibitor ABT-737. Genetic loss of Bcl-X_L_ or treatment of macrophages with ABT-737 reduced the generation of infectious VACV. These data show that Mcl-1 is dispensable for the regulation of apoptosis during infection with different large DNA viruses, either because the viruses replace its function (in fibroblasts and epithelial cells) or because the pro-apoptotic activity generated by the infection appears not to be blocked by it (in macrophages). Bcl-X_L_, on the other hand, can be important to maintain survival of virus-infected cells, and its activity can determine outcome of the infection.

Apoptosis of the infected cell is a defence strategy of higher organisms against viral infection. Cells have the ability to sense the presence of the virus, and this can often initiate apoptosis. If an infected cell dies by apoptosis, then viral replication is likely precluded. This interpretation of the role of apoptosis during viral infection is supported by the presence of genes coding for anti-apoptotic proteins in many viruses, ranging from baculoviruses (infecting insect cells^1^) to complex DNA viruses infecting human cells such as poxviruses.^2^

To undergo apoptosis upon viral infection, the host cell has to be able to recognize the infection and to initiate the apoptosis system. Prominently, recognition occurs by pattern recognition receptors of innate immunity, which most often recognize viral nucleic acids differing in certain structural characteristics from host cell nucleic acids (for review, see Hornung *et al.*^3^). How this recognition machinery is linked to the apoptosis apparatus is less clear. The transfection of RNA that mimics viral RNA and that activates cytosolic helicases has been shown to activate the mitochondrial apoptosis pathway, inducing several pro-apoptotic proteins of the Bcl-2-family. In that study, the pro-apoptotic Bcl-2-family protein Noxa was required for apoptosis.^4^

Cell survival and apoptosis are regulated by the Bcl-2 family of proteins. Within the Bcl-2 family, the class of BH3-only proteins (including Bim, Bid, Puma, Noxa and Bmf) triggers the activation of the ‘executor' proteins Bax and Bak, directly or indirectly. Bax and Bak can oligomerize and release cytochrome *c* from the mitochondria into the cytosol, where it activates caspases, leading to apoptosis. The anti-apoptotic Bcl-2-family proteins (Bcl-2, Bcl-X_L_, Bcl-w, Mcl-1 and A1) inhibit this process by binding to both BH3-only proteins and Bax/Bak with varying affinity.^5, 6^ The decision to undergo apoptosis is taken through the balance of pro- and anti-apoptotic Bcl-2-family proteins. Apoptosis may be triggered by upregulation or in some cases activation of BH3-only proteins, or by the loss of anti-apoptotic proteins.

Both Poxviruses and herpes viruses are large, double-stranded DNA viruses with very different cell biology. Both types carry genes whose products resemble mammalian anti-apoptotic proteins.^7^ Intriguingly, poxviruses have many genes that from their primary structure are not recognizable as Bcl-2 homologues and that do necessarily not act by inhibiting apoptosis but have a very similar spatial structure to Bcl-2 (see for instance Neidel *et al.*^8^).

Although the mammalian Bcl-2-like anti-apoptotic proteins have an overall similar, conserved structural fold and all can block apoptosis by binding to pro-apoptotic Bcl-2-family proteins, they substantially differ in terms of this binding affinity as well as their biological function. Deletion of individual anti-apoptotic Bcl-2-proteins in mice produces rather different phenotypes, ranging from male sterility (deletion of Bcl-w) and cell death of specific populations in various organs (loss of Bcl-2) to embryonic lethality (loss of Bcl-X or Mcl-1).^9^

Bcl-X_L_ and Mcl-1 appear to have complementary functions. In molecular terms, both proteins are thought to bind to and inhibit pro-apoptotic Bak in resting cells.^10^ A number of studies have compared the importance of Bcl-X_L_ and Mcl-1 for survival of specific populations and situations. The loss of Mcl-1 leads to spontaneous apoptosis of haematopoietic stem cells as well as of all haematopoietic lineages tested, with the exception of macrophages.^11, 12^ Bcl-X_L_ deficiency causes embryonic lethality very likely due to neuronal apoptosis. In a number of studies, it was found consistently that loss of Mcl-1 reduced survival of the affected cell populations; this was the case for regulatory T cells,^13^ haematopoietic stem/progenitor cells,^14^ plasma cells^15^ and NK cells^16^ as well as malignant cells, as shown for myc-driven^17^ and thymic lymphoma.^18^ Where Bcl-X_L_ was deleted in the same cell populations, its loss had no effect.^13, 14, 15, 18^ Melanoma cells have been shown to undergo apoptosis upon the sole loss of Mcl-1.^19^ Non-transformed skin cells, however, underwent apoptosis only upon RNAi-mediated loss of both Bcl-X_L_ and Mcl-1.^20^

We here tested whether Bcl-X_L_ and Mcl-1 have different roles during the infection of mammalian cells with large DNA viruses. We found that, in fibroblasts and epithelial cells, all three viruses tested (MVA and VACV are very similar poxviruses, murine cytomegalovirus (MCMV) is a herpes virus) could replace Mcl-1 function and failed to induce apoptosis. Macrophages, on the other hand, showed substantial apoptosis when infected by any of the three viruses. Surprisingly, Mcl-1, which is critical for survival in many situations, also had no role in protecting macrophages against virus-induced apoptosis. Bcl-X_L_, on the other hand, did contribute to the survival of infected cells, and its activity contributed to the regulation of survival of infected macrophages.

## Results

### Infection of epithelial cells and fibroblasts by MVA leads to a loss of Mcl-1 protein but viruses can replace Mcl-1 functionally

**Figure 1:**
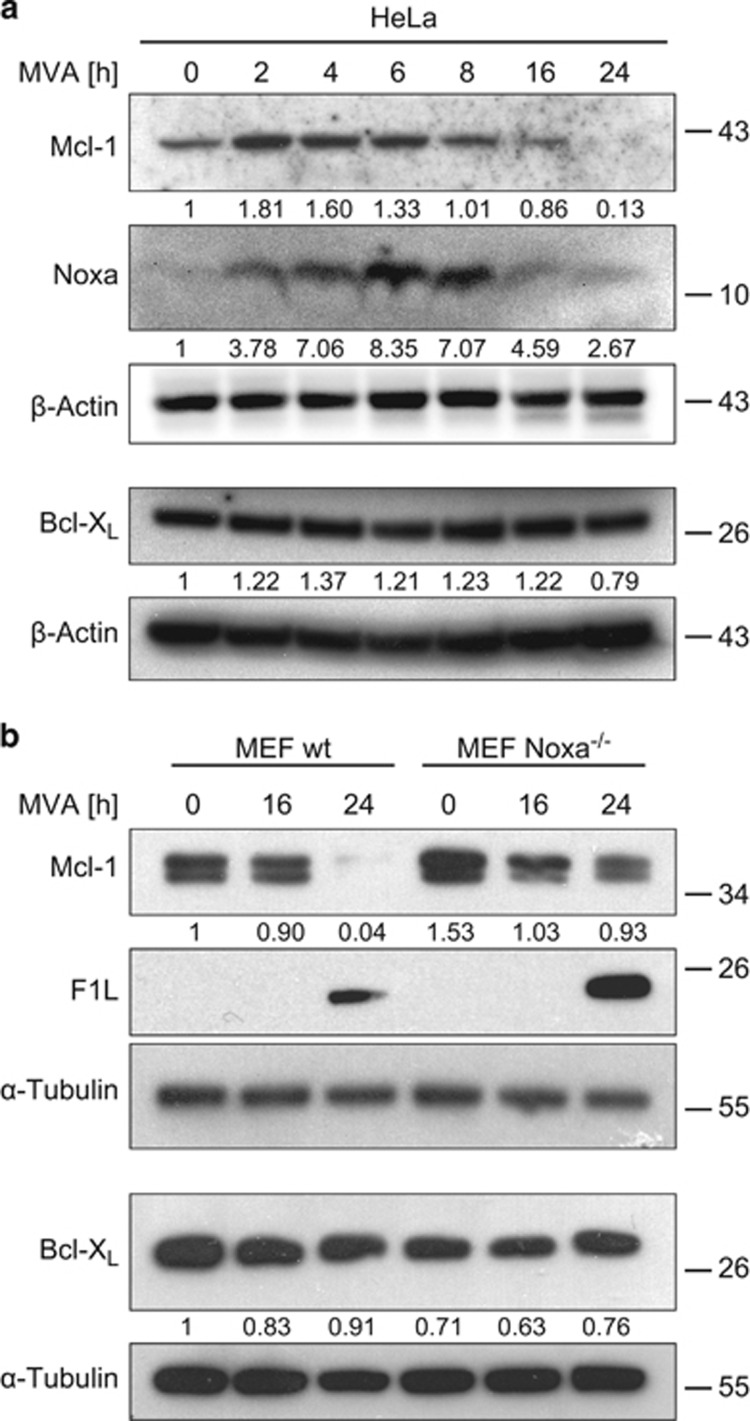
(**a**) Infection of HeLa cells with MVA leads to the upregulation of Noxa and the loss of Mcl-1 protein. HeLa cells were infected with MVA (MOI=10) for the times indicated. Protein levels were determined by western blotting. Signals were quantified by densitometry and changes in Mcl-1, Noxa or Bcl-X_L_/*β*-Actin ratios normalized to the uninfected control are shown below the blots. Data are representative of at least four independent experiments. (**b**) MVA infection leads to a reduction in Mcl-1 levels but has no effect on Bcl-X_L_ levels in MEFs. MEF wt or Noxa^−/−^ cells were infected with MVA (MOI=10) for 16 or 24 h. Protein levels were assessed by western blotting. Signals were quantified by densitometry and changes in Mcl-1 or Bcl-X_L_/α-Tubulin ratios normalized to the uninfected wt control are shown below the blots. Data are representative of at least six independent experiments

Infection of HeLa human epithelial cells with MVA or MVAΔF1L caused the loss of anti-apoptotic Mcl-1 after about 8–16 h post infection (Figure 1a and Supplementary Figure S2A). This loss was not seen at the mRNA level (where there was even some increase during infection (Supplementary Figure S1A and B)) and may in part be due to the induction of the Mcl-1-antagonist Noxa, which can trigger the degradation of Mcl-1,^21^ and which is known to be upregulated in the response of HeLa cells to MVA infection (Figure 1a).^22^ Noxa was lost again at later stages of infection, possibly indicating reduced synthesis or co-degradation with Mcl-1. A similar loss of Mcl-1 was observed in mouse embryonic fibroblasts (MEFs), and the loss of Mcl-1 was less profound in MEFs deficient in Noxa (Figure 1b and Supplementary Figure S2B), suggesting that Noxa contributes to the disappearance of Mcl-1. Bcl-X_L_ levels were unaffected by MVA infection. The Bcl-2-like MVA-protein F1L^23^ was detectable during infection (Figure 1b).

**Figure 2:**
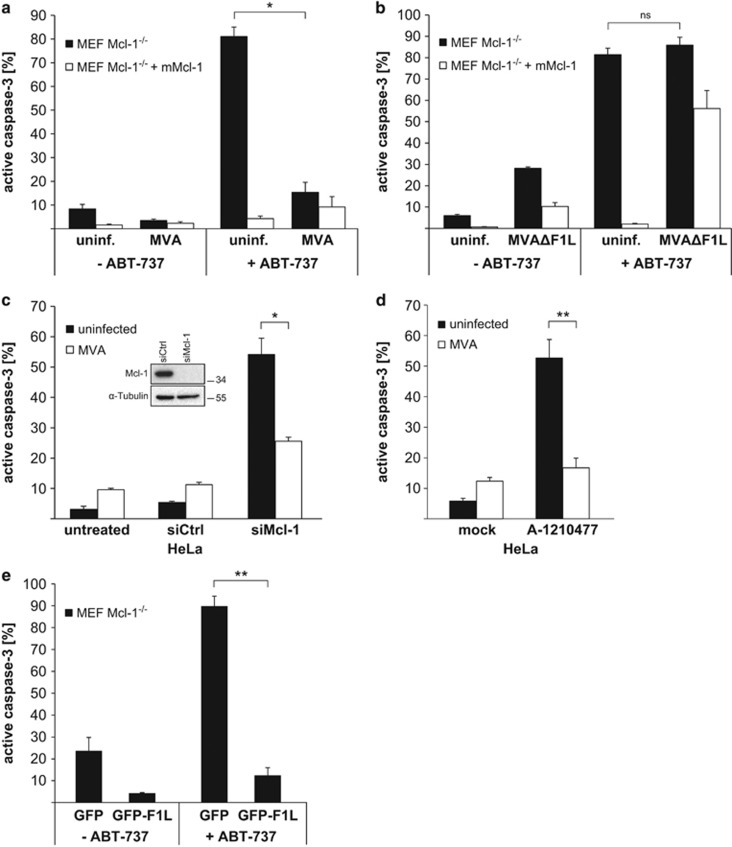
(**a** and **b**) Infection with MVA but not MVAΔF1L protects Mcl-1-deficient MEF cells against the Bcl-2/Bcl-X_L_-antagonist ABT-737. MEF Mcl-1^−/−^ cells or MEF Mcl-1^−/−^ reconstituted with murine Mcl-1 were infected with MVA (**a**) or MVAΔF1L (**b**) (MOI=10) for 16 h. Then, 1 μM ABT-737 was added for additional 4 h where indicated. Apoptosis induction was measured as the percentage of cells positive for active caspase-3 by flow cytometry. Data show means/S.E.M. of three independent experiments (*P=0.013 (**a**); ns, *P*=0.25 (**b**); ns, not significant; two-tailed paired *t*-test). (**c**) MVA protects against cell death in HeLa induced by knockdown of Mcl-1. HeLa cells were infected with MVA (MOI=10) for 18 h and simultaneously transfected with siRNA directed against Mcl-1 (siCtrl, non-specific siRNA). Apoptosis induction was measured as the percentage of cells positive for active caspase-3 by flow cytometry and protein levels were assessed by western blotting. Data show means/S.E.M. of four independent experiments (*P=0.02; two-tailed paired *t*-test). (**d**) MVA protects against cell death in HeLa induced by the Mcl-1 inhibitor A-1210477. HeLa cells were infected with MVA (MOI=10) for 18 h and afterwards treated with the Mcl-1 inhibitor A-1210477 (10 *μ*M) for 4 h. Apoptosis induction was measured as the percentage of cells positive for active caspase-3 by flow cytometry. Data are representative of three independent experiments (**P=0.007; two-tailed paired *t*-test). (**e**) Expression of MVA-F1L protein protects against ABT-737 induced apoptosis in Mcl-1-deficient MEF cells. MEF Mcl-1^−/−^ cells were transiently transfected with EGFP-F1L expression plasmid or empty vector for 24 h and 1 *μ*M ABT-737 was added for another 3 h where indicated. Apoptosis induction was measured as the percentage of cells positive for active caspase-3 by flow cytometry (only cells positive for GFP were selected for the analysis (efficiency of transfection was at least 55%)). Data show means/S.E.M. of three independent experiments (**P=0.004; two-tailed paired *t*-test)

In the absence of F1L, MVA induces apoptosis upon infection of HeLa and MEF cells (Supplementary Figure S1C and D),^22^ suggesting that a molecular function of F1L is the replacement of Mcl-1. We tested this possibility by inactivating Bcl-2 and Bcl-X_L_ with the small molecule inhibitor ABT-737. In cells expressing sufficient levels of Mcl-1, ABT-737 on its own fails to induce apoptosis, but the loss of Mcl-1 sensitizes most cells to ABT-737-induced apoptosis.^24^ Unlike wt cells, Mcl-1-deficient MEFs died rapidly upon treatment with ABT-737 while re-expression of Mcl-1 protected the cells. MVA infection however protected Mcl-1-deficient cells against ABT-737 (Figure 2a).

Infection with MVA deficient in F1L (MVAΔF1L) induced apoptosis in HeLa and MEFs as reported earlier^23^ (Supplementary Figure S1C and D) and was unable to protect Mcl-1-deficient MEFs against ABT-737 (Figure 2b). In HeLa cells, the sole targeting of Mcl-1 by RNAi induced apoptosis, and this was at least partially reversed by infection with MVA (Figure 2c). Additionally, targeting Mcl-1 with the small molecule inhibitor A-1210477^25^ in HeLa cells led to cell death, which could also be inhibited by preceding infection with MVA (Figure 2d). The ability to replace Mcl-1 was further confirmed by transfection of Mcl-1-deficient MEFs with an expression construct of MVA-F1L. Expression of F1L protected the cells against ABT-737-induced apoptosis (Figure 2e). During MVA infection of HeLa and MEFs, therefore Mcl-1 is lost but is replaced by viral F1L.

**Figure 3:**
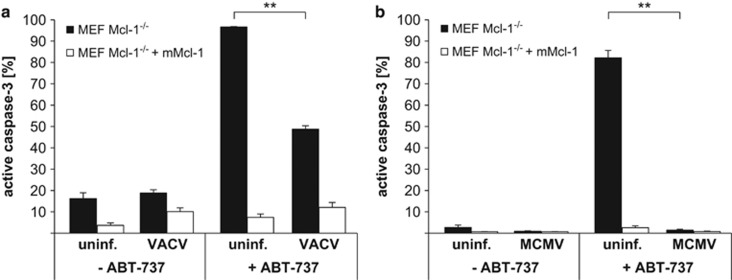
(**a** and **b**) Infection with VACV or MCMV protects Mcl-1-deficient MEF cells against ABT-737. MEF Mcl-1^−/−^ cells and MEF Mcl-1^−/−^ reconstituted with mMcl-1 were infected with VACV (**a**) or MCMV (**b**) (MOI=10) for 16 h. Then, 1 *μ*M ABT-737 was added for additional 4 h where indicated. Apoptosis induction was measured as the percentage of cells positive for active caspase-3 by flow cytometry. Data show means/S.E.M. of three independent experiments (**P=0.0013 (**a**); **P=0.0016 (**b**); two-tailed paired *t*-test)

VACV^26^ and MCMV^27^ have likewise been found to have anti-apoptotic activity. We tested whether infection with these viruses also replaced Mcl-1 function. Infection of Mcl-1-deficient MEFs with VACV provided substantial protection against ABT-737 (Figure 3a), while MCMV-infected cells were completely protected (Figure 3b). This suggests that the expression of Mcl-1-like activity is a broader effect of viral infection. The loss of Mcl-1 can also be seen during VACV infection in MEF and HeLa cells (Supplementary Figure S2). Infection with MCMV did not alter expression of Mcl-1 in HeLa and even induced Mcl-1 expression in MEFs. Although not generally applicable, the loss of Mcl-1 may be a more general defence mechanism of mammalian cells attempting to undergo apoptosis upon viral infection. However, at least some viruses have evolved proteins that replace Mcl-1 function, and a downregulation of Mcl-1 by an infected cell is futile.

### Survival of MVA-infected macrophages is independent of Mcl-1 but depends on Bcl-X_L_

Upon infection of the organism, viruses are often primarily taken up by macrophages. Upon i.v. infection of mice with MVA for instance viral gene expression in spleen is primarily found in macrophages and dendritic cells.^22^ Similarly, MCMV has been found to infect monocytes and induce their differentiation to macrophages before spreading of the virus.^28^ In terms of anti-apoptotic proteins, macrophages are unique among the tested haematopoietic cells in that they do not depend on Mcl-1 for survival.^12^ This suggested that the viral strategy of replacing Mcl-1-function may not be able to protect macrophages during infection.

Mcl-1- or Bcl-X-deficient mice are not viable. To test for the relative roles of Mcl-1 and Bcl-X_L_ in macrophages during viral infection, we used cells derived from mice carrying the respective alleles flanked by cre-lox-recognition sites together with Cre under the LysM promoter. In this system, Cre is expressed during macrophage differentiation and should cause the deletion of Mcl-1 or Bcl-X_L_. As cellular system, we utilized a model where mature mouse macrophages can be differentiated from committed macrophage progenitor cells that are ‘conditionally transformed' with the oncogene Hoxb8, fused to the ligand-binding domain of the oestrogen receptor. In the presence of oestrogen, Hoxb8 is active and drives the expansion of progenitor cells, and in this way lines of committed macrophage progenitors can be established. When oestrogen is removed from the culture medium cells start to differentiate and after 7–10 days express the markers of mature murine macrophages.^29^

Progenitor lines of either background (LysM-Cre/Mcl-1^flox/flox^ or LysM-Cre/Bcl-X_L_^flox/flox^) were generated and expanded. Since the cells were from different genetic crosses, we used wt cells isolated from littermates of each breeding. Cells were induced to differentiate, and maturation was tested by staining for the markers CD11b (myeloid marker) and F4/80 (macrophage-specific marker) on their surface. No differences in expression of these markers between the gene-deficient cells and the corresponding wt cells were noted (Supplementary Figure S3), and there was no obvious cell death during differentiation in culture (not shown). Bcl-X_L_ was expressed at somewhat reduced levels in progenitors carrying the targeted allele, indicating that Cre is expressed at low levels or only in some cells up to this progenitor stage. A reduction in Mcl-1 was already observed in progenitors carrying the loxP-flanked Mcl-1-allele (Supplementary Figure S4). Differentiation of macrophages decreased expression of the respective gene to levels that were very low, sometimes undetectable, indicating Cre-mediated loss during differentiation (Supplementary Figure S4). The results confirm that Mcl-1 is not essential for survival of macrophages and demonstrate that Bcl-X_L_ is likewise dispensable for the development of macrophages from these progenitors.

**Figure 4:**
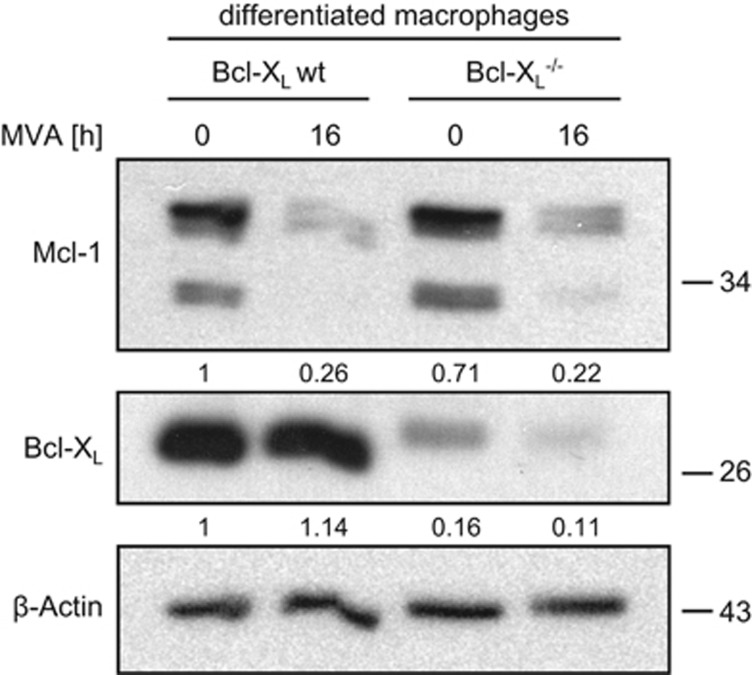
MVA infection leads to a reduction in Mcl-1 levels but has no effect on Bcl-X_L_ levels in differentiated macrophages, independently of Bcl-X_L_ status. The bands detected in the Bcl-X_L_^−/−^ cells are probably due to incomplete deletion of the gene by the LysM-Cre transgene. Macrophages (wt or Bcl-X_L_^−/−^) were infected with MVA (MOI=10) for 16 h. Protein levels were assessed by western blotting. Signals were quantified by densitometry and changes in Mcl-1 or Bcl-X_L_/*β*-Actin ratios normalized to the uninfected wt control are shown below the blots. Data are representative of at least eight independent experiments

**Figure 5:**
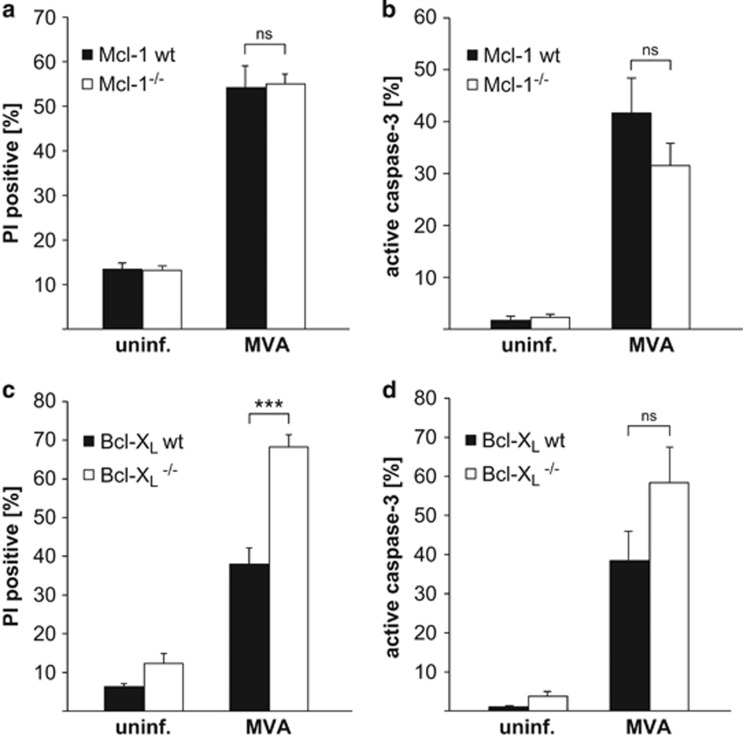
Infection with MVA induces cell death in macrophages independent of Mcl-1 deficiency (**a** and **b**), but is enhanced when Bcl-X_L_ is absent (**c** and **d**). On day 7 of differentiation, macrophages wt, Mcl-1^−/−^ (**a** and **b**) or wt, Bcl-X_L_^−/−^ (**c** and **d**) were infected with MVA (MOI=10) for 16 h. Cell death was measured as the percentage of cells positive for PI (**a** and **c**) and apoptosis induction as the percentage of cells positive for active caspase-3 (**b** and **d**) by flow cytometry. Data show means/S.E.M. of three (**a**), five (**b**) or six (**c** and **d**) independent experiments (ns, *P*=0.88 (**a**); ns, *P*=0.24 (**b**); ***P=0.0002 (**c**); ns, *P*=0.12 (**d**); ns, not significant; two-tailed unpaired *t*-test)

Infection of mature macrophages with MVA led to a loss of Mcl-1 but not Bcl-X_L_, similar to the events observed in the above experiments in epithelial (HeLa) and MEF cells (Figure 4). As we have reported earlier,^22^ MVA infection induces substantial apoptosis in macrophages (Figure 5). Mcl-1-deficient macrophages showed no enhanced apoptosis when infected with MVA, compared with the corresponding wt cells (Figures 5a and b). However, Bcl-X_L_ deficiency clearly enhanced sensitivity of macrophages to MVA-induced apoptosis (Figures 5c and d). Mcl-1 is thus not involved in the regulation of survival and apoptosis during MVA infection in macrophages, but Bcl-X_L_ has a role in keeping infected macrophages alive.

In epithelial cells and HeLa cells, MVA infection induces an interferon response, which through the induction of the pro-apoptotic Bcl-2-family protein Noxa contributes to apoptosis induction (which is then blocked by F1L^22^). We therefore tested macrophages deficient in these genes and found a small protection against MVA infection by Noxa deficiency, but no difference in cells where type I interferon signalling is impossible (IFNAR-deficient cells) (Supplementary Figure S5). Interferon signalling therefore appears to have no role in the apoptotic response.

### Bcl-X_L_ contributes to apoptosis resistance during infection with VACV and MCMV

**Figure 6:**
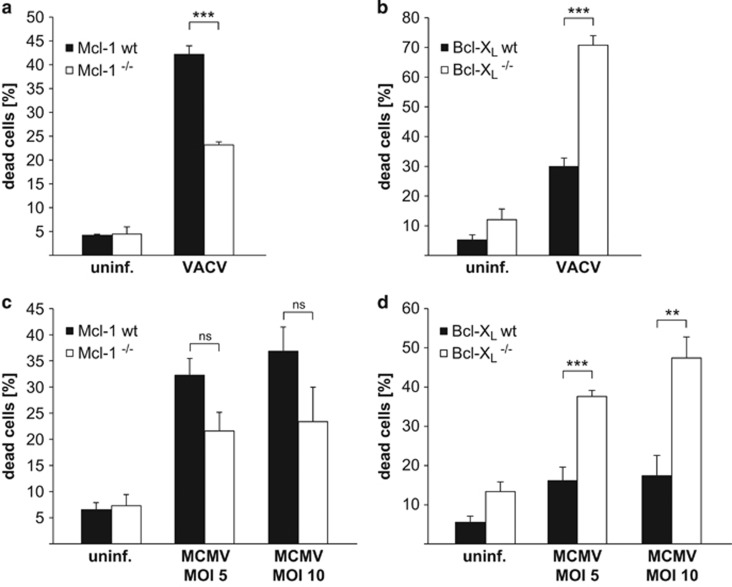
Infection with VACV or MCMV leads to enhanced cell death in Bcl-X_L_^−/−^ macrophages but Mcl-1^−/−^ macrophages are similarly or less sensitive to apoptosis compared with wt. On day 7 of differentiation, macrophages wt or Mcl-1^−/−^ (**a** and **c**) or wt or Bcl-X_L_^−/−^ (**b** and **d**) were infected with VACV for 16 h (MOI=10) (**a** and **b**) or MCMV for 24 h (MOI=5 or 10) (**c** and **d**). Cell death was measured as the percentage of cells stained positive for the Far Red dye of the Dead Cell Stain Kit. Data show means/S.E.M. of three (**a**), four (**b** and **c**) or five (**d**) experiments (***P=0.0006 (**a**); ***P=0.00007 (**b**); ns, *P*=0.07 (**c**, MOI=5); ns, *P*=0.15 (**c**, MOI=10); ***P=0.0005 (**d**, MOI=5); **P=0.004 (**d**, MOI=10); ns, not significant; two-tailed unpaired *t*-test)

We next tested whether this role of Bcl-X_L_ is limited to MVA infection or also found during infection with VACV and MCMV. We found that the response to infection with these viruses was similar: Bcl-X_L_-deficient macrophages showed an increased apoptotic response upon infection whereas Mcl-1-deficient macrophages did not (Figure 6, infection efficiencies shown in Supplementary Figure S6). Indeed, Mcl-1-deficient macrophages were consistently less sensitive to apoptosis induced by either virus (Figures 6a and c). Infection with VACV induced the loss of Mcl-1 in macrophages, as we had observed during infection with MVA (Supplementary Figure S7A). No such effect was observed during infection of macrophages with MCMV (Supplementary Figure S7B).

**Figure 7:**
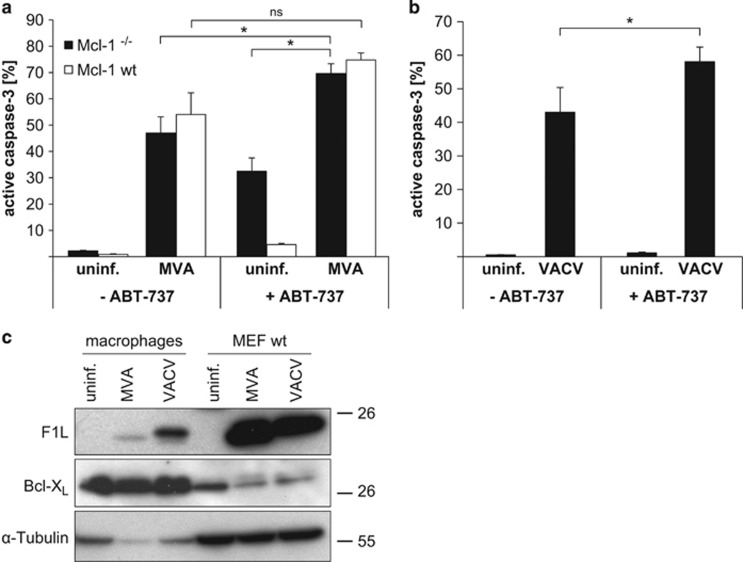
(**a** and **b**) Infection with MVA or VACV sensitizes macrophages to ABT-737. (**a**) Wt or Mcl-1^−/−^ macrophages were infected with MVA (MOI=10) for 16 h. Then, 1 μM ABT-737 was added for additional 4 h where indicated. Apoptosis induction was measured as the percentage of cells positive for active caspase-3 by flow cytometry. Data show means/S.E.M. of three independent experiments (*P≤0.05; ns, *P*=0.13; ns, not significant; two-tailed paired t-test). (**b**) Wt macrophages were infected with VACV (MOI=10) for 16 h. Then, 1 μM ABT-737 was added for additional 4 h where indicated. Apoptosis induction was measured as the percentage of cells positive for active caspase-3 by flow cytometry. The wt macrophages from the Bcl-X_L_ cross were used here. Data show means/S.E.M. of five independent experiments (*P=0.048; two-tailed paired *t*-test). (**c**) Only very little F1L protein is detectable during infection of macrophages with MVA or VACV. Differentiated macrophages or MEF wt cells were infected with MVA or VACV (MOI=10) for 16 h. Same amounts of protein (80 μg) were loaded, and specific protein levels were assessed by western blotting. *α*-Tubulin was used as a standard loading control and Bcl-X_L_ as a specific control for mitochondria. Data are representative of at least four independent experiments

The lack of a role of Mcl-1 in macrophages suggested that the viral strategy of replacing Mcl-1, which had been apparent in fibroblasts, may also not be successful in macrophages. When we tested for an Mcl-1-replacing activity we found it during neither MVA nor VACV infection of macrophages. In MVA-infected macrophages, ABT-737 even enhanced virus-induced apoptosis, regardless whether Mcl-1 was present or not (Figure 7a). Since ABT-737 blocks Bcl-X_L_ function, this enhanced apoptosis is probably the result of this blockade. Nevertheless, Mcl-1 did have a role in protecting macrophages against ABT-737 (compare apoptosis induced by ABT-737 in wt and Mcl-1-deficient cells, Figure 7a), yet there was no protection by MVA infection in Mcl-1-deficient cells. ABT-737 also enhanced apoptosis induced by VACV in wt macrophages (Figure 7b), confirming the central role of Bcl-X_L_ rather than Mcl-1 (which is not sensitive to ABT-737) during VACV infection.

Although this observation confirmed the respective roles of Mcl-1 and Bcl-X_L_ found in the gene-deficient cells, it was surprising since the results in HeLa and MEFs described above had shown that MVA-F1L (and an unidentified activity in VACV, very likely VACV F1L) is able to replace Mcl-1 and to protect against ABT-737. When we tested for F1L expression, we however found that infected macrophages expressed only very little, sometimes undetectable amounts of F1L during infection with MVA or VACV (Figure 7c). Although the molecular basis is unclear, this suggests that reduced expression of anti-apoptotic F1L during macrophage infection contributes to the strong pro-apoptotic activity of MVA and VACV infection.

**Figure 8:**
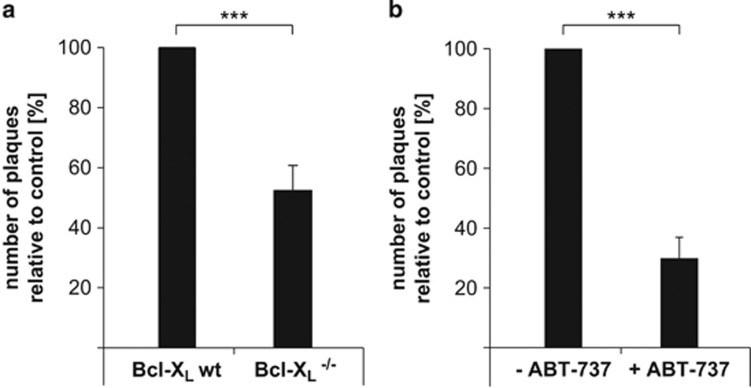
(**a**) The amount of released infectious viral particles is reduced in Bcl-X_L_^−/−^ macrophages compared with wt during VACV infection. On day 7 of differentiation, wt or Bcl-X_L_^−/−^ macrophages were infected with VACV (MOI=10) for 16 h. Plaque assay was performed to measure infectious virus particles using infected cells plus supernatant at 1 : 1000 dilution. The number of plaques was counted. Number of plaques from wt cells was set to 100%. Data show means/S.E.M. of eight independent experiments with duplicate samples each (***P=0.00005; two-tailed unpaired *t*-test). (**b**) Treatment with ABT-737 reduced the amount of released infectious viral particles during VACV infection in macrophages. On day 7 of differentiation, wt macrophages were infected with VACV (MOI=10) for 16 h. Then, 1 μM ABT-737 was added for additional 4 h where indicated. Plaque assay was performed to measure infectious virus particles using infected cells plus supernatant at 1 : 1000 dilution. The number of plaques was counted. Number of plaques from untreated cells was set to 100%. Data show means/S.E.M. of five independent experiments with duplicate samples each (***P=0.0006; two-tailed paired *t*-test)

Apoptosis is typically viewed as a defence mechanism against viral infection. If apoptosis has this role in macrophage infection, then the observation that the loss of Bcl-X_L_ function, which enhances virus-induced macrophage apoptosis, predicts that loss of Bcl-X_L_ in macrophages also reduces the generation of infectious virus. We tested this hypothesis by measuring the generation of infectious VACV upon infection of wt *versus* Bcl-X_L_-deficient macrophages (MVA cannot replicate efficiently in mouse cells). Indeed, Bcl-X_L_-deficient macrophages released only about half the number of infectious viral particles of infected wt macrophages (Figure 8a). Treatment of wt macrophages with ABT-737 reduced the production of infectious VACV even more strongly, to about 30% (Figure 8b). Apoptosis of infected macrophages is thus a factor in reducing the production of infectious VACV, and apoptosis may help limit the initial replication of the virus. Treatment with Bcl-X_L_ antagonists has the capacity to help reducing the generation of virus during macrophage infection.

## Discussion

Our results identify a differential role of the anti-apoptotic proteins Bcl-X_L_ and Mcl-1 during infection of mammalian cells with large DNA viruses. Mcl-1, in contrast to its essential role in many other situations, has no role in the regulation of survival during infection with MVA/VACV or MCMV. In epithelial and fibroblast cells, this is very likely due to the expression of viral anti-apoptotic proteins, in the case of MVA due to F1L. In macrophages, Mcl-1 has no critical role in steady-state survival, nor has Bcl-X_L_. Mcl-1 also has no role in controlling macrophage apoptosis in response to infection with these viruses. However, Bcl-X_L_ does provide a measure of protection, and its loss or its inactivation with ABT-737 increases the apoptotic response and reduces the replication of VACV. We hasten to add that we only used one particular strain of VACV and of MCMV and can well picture that other strains may have different pro- or anti-apoptotic potential in a given cell type.

Of the Bcl-2-homologues, Mcl-1 has a particularly critical function in a number of cell types, especially in the immune system (while no role of Bcl-X_L_ in many situations is apparent, see Introduction). Why Mcl-1 has this important role is not clear but this may be linked to the strong regulation of Mcl-1 by extrinsic stimuli (such as cytokines, see for instance Sathe *et al.*^16^) and major signalling pathways (such as the GSK-3 pathway^30^). Mcl-1 can have a very rapid turnover, and a number of enzymes are known to regulate its proteasomal degradation.^31^ Mcl-1 may therefore be the main anti-apoptotic Bcl-2-family member that responds to stimuli from the outside.

Indeed, Mcl-1 does respond to viral infection. Mcl-1 protein (but not mRNA) is reduced or lost upon infection with MVA/VACV. This is in part due to the induction of Noxa, which in itself is an interferon-response gene that is induced in antiviral defence, for instance during infection with MVA (Eitz Ferrer *et al.*^22^ and this study).

In the case of MVA, our data show that the viral protein F1L can fulfil the molecular function of Mcl-1. Without F1L, MVA is known to induce apoptosis, and F1L could directly replace Mcl-1 in Mcl-1-deficient cells. VACV has a very similar F1L orthologue, which has been demonstrated to bind Bak (a molecular function of Mcl-1 and Bcl-X_L_^32^). Infection with MCMV could replace Mcl-1 function in Mcl-1-deficient MEFs, suggesting that this is a common viral strategy, and implying that the loss of Mcl-1 protein or function is a common aspect of host cell defence against viral infection.

It has been reported earlier that MVA,^22^ VACV^33^ and MCMV^34^ all can induce apoptosis in macrophages (although there appeared to be some strain variation with VACV derivatives^35^), while no or little apoptosis is induced (and apoptosis is typically inhibited) in fibroblasts or epithelial cells. The reason for this is molecularly unclear. One point may be that recognition and response to viral components are stronger in macrophages as professional immune cells. During MVA infection, recognition of viral RNA and the subsequent interferon response account for part of the apoptotic response.^22^ This pro-apoptotic activity of viral infection may simply be stronger in macrophages.

However, two factors are also likely to contribute to the higher sensitivity of macrophages. First, many cells are more dependent on Mcl-1 than macrophages. Many cell lines die upon RNAi against Mcl-1 (see HeLa in Figure 2c), and although this may be linked to the malignant transformation of the cells, it is remarkable that macrophages are the only major lineage of immune cells that do not require Mcl-1. In macrophages, apoptotic stimuli may therefore rely more heavily on pathways not involving Mcl-1, and viral infection may be one such stimulus. Second, viruses appear to have difficulty in establishing protection against apoptosis in macrophages. Unlike during infection of fibroblasts, MVA was unable to protect Mcl-1-deficient macrophages against ABT-737; a sensitization to ABT-737 was even seen during VACV infection. Consistently, very little F1L could be detected during infection with MVA or VACV despite productive infection (in the case of VACV). Although it is unclear how this is achieved, a low expression of anti-apoptotic proteins may contribute to apoptosis induction in macrophages.

However, Bcl-X_L_ turned out to be an important pro-survival factor in macrophages. In most situations and cell types where this has been directly compared, Bcl-X_L_ was much less important than Mcl-1 in keeping the cells alive (see Introduction). The very clear apoptosis-enhancing effect we observed in macrophages upon the loss of Bcl-X_L_ was therefore surprising. The findings suggest that during viral infection of macrophages a pro-apoptotic activity is generated that is to a substantial degree blocked by Bcl-X_L_ but not by Mcl-1. What the nature of such a stimulus could be, and how it is transmitted to the mitochondria and the Bcl-2-family of proteins is unclear. There are some pro-apoptotic proteins that can be blocked by Bcl-X_L_ but no by Mcl-1; most notably, some of the pro-apoptotic Bcl-2-family group of BH3-only proteins bind much more strongly to Bcl-X_L_ than to Mcl-1. Bad, for instance has an affinity for Bcl-X_L_ that is at least 100 000 times higher than for Mcl-1; for Bmf, the difference is about 100, for Bid about 25-fold (measuring binding of the BH3 domains^36^). It is therefore at least conceivable that such pro-apoptotic molecules are involved. We did test macrophages deficient in Bim and Bmf; however, while Bim-deficient macrophages showed small protection against MVA-induced apoptosis (as reported earlier^22^) additional loss of Bmf had no effect (data not shown).

The development of small molecules that specifically target individual proteins of the apoptosis apparatus in recent years has helped understand apoptosis and may contribute to future treatment of disease. ABT-737 is a molecule that binds to and inhibits Bcl-2, Bcl-X_L_ and Bcl-w;^37^ more specific molecules targeting only individual Bcl-2-family members are also available. The enhancement of virus-induced apoptosis in macrophages by ABT-737 confirms a pro-survival role of Bcl-X_L_ (and perhaps Bcl-2) during viral infection. Although the enhanced apoptosis by deletion of Bcl-X_L_ or by treatment with ABT-737 can explain the reduction in progeny generation observed, it has also been proposed that such manipulation may enhance autophagy, and enhanced autophagy might add to viral clearance and therefore also contribute to the reduction in virus generation. It has to be said however that this issue cannot be considered clarified at present. An apoptosis-independent contribution of anti-apoptotic Bcl-2 proteins to autophagy has been disputed.^38^ More recently, this has been challenged by demonstrating that ABT-737 can induce autophagy in the absence of Bax and Bak,^39^ but this has in a very recent study been suggested to be due to effects on components other than Bcl-2-like proteins.^40^ This question therefore has to await further clarification.

It may be presumptuous at this stage to suggest that such inhibitors may be used in clinical settings, but a clear understanding of the pro- and anti-apoptotic processes occurring in infectious disease and in the different types of infected cells will help understand the biology and may indeed at some stage also provide a new handle on therapeutic approaches.

## Materials and Methods

### Cell lines and culture conditions

MEFs wt, deficient for Noxa (Noxa^−/−^, immortalized by the 3T3 method, kindly provided by Christoph Borner, Freiburg) or Mcl-1 (Mcl-1^−/−^, immortalized with SV40, and the same cells reconstituted with mouse Mcl-1, kindly provided by Ulrich Maurer, Freiburg) were cultured in Dulbecco's Modified Eagle Medium (DMEM, #41965062, Thermo Scientific, Reinach, Switzerland), supplemented with 10% fetal calf serum (FCS), antibiotics (100 U/ml penicillin G and 100 *μ*g/ml streptomycin sulphate) and 50 *μ*M 2-mercaptoethanol. HeLa cells were grown in DMEM containing 10% FCS and antibiotics as above. BSC-40 cells were grown in DMEM containing 5% FCS, 1% glutamine and antibiotics as above.

Hoxb8 macrophage progenitors were derived from bone marrow of mice homozygous for a conditional (loxP-flanked) Bcl-x,^41^ Mcl-1,^42^ Bim or Bim and Bmf allele and heterozygous for the LysM-cre-allele,^43^ or from Noxa^−/−^ or IFNAR^−/−^ Mx1 mice (kindly provided by Peter Stäheli, Freiburg). All mice were backcrossed on the C57Bl/6 background but since they came from different breeding programmes the corresponding wt cells were for all lines derived at the same time and from littermates. Polyclonal precursor cell lines were established by retroviral transduction of Hoxb8 and selection in the presence of granulocyte macrophage colony-stimulating factor (GM-CSF).^29^ After selection macrophage precursor cell lines were cultured in RPMI-1640 medium (#F1415, Merck Millipore, Darmstadt, Germany) containing 10% heat-inactivated FCS (#10270-106, Thermo Scientific), antibiotics, 1 : 100 diluted supernatant of GM-CSF producing B16 cells and 1 *μ*M *β*-estradiol. Macrophage differentiation was induced by removal of *β*-estradiol, followed by culture for 7 days in medium containing 1% GM-CSF. All cultures were incubated under standard culture conditions (37 °C, 5% CO_2_).

To assess the differentiation status, progenitors or day 7 or 8 differentiated macrophages were washed in PBS and incubated with Fc block antibody (#553142, BD, Heidelberg, Germany) for 20 min before F4/80 and CD11b staining (anti-F4/80-Alexa Fluor 647, #MCA497A647, AbD Serotec, Munich, Germany; anti-CD11b-PE, #12-0112-82, eBioscience, Frankfurt, Germany) for 20 min on ice. Cells were analysed by flow cytometry using an FACS Calibur (Becton Dickinson).

### Virus infections

For Modified Virus Ankara (MVA) or Vaccinia virus (VACV WR; kindly provided by Peter Aichele, Freiburg) infections, MEF and HeLa cells were seeded at 1.5 × 10^5^ cells/well in 6-well plates the day before infection. At least 30 min before infection, medium was exchanged for 600 *μ*l of infection medium without antibiotics and 2% FCS. MVA or MVAΔF1L^22^ was diluted in infection medium, vortexed and sonicated for 1 min. Cells were infected with an MOI of 10 in 600 *μ*l infection medium for indicated times.

Macrophages differentiation was started with 8 × 10^4^ progenitor cells/well in a 6-well plate 7 days before the infection. Infection was conducted as above but in 1 ml volume.

Infection efficiency of VACV was determined by infecting cells with VACV-GFP (VACV B5R-EGFP, kindly provided by Peter Aichele, Freiburg) for 5 h and performing flow-cytometry analysis of the expression of viral GFP.

Infections with MCMV were performed as above. Addition of the virus was followed by centrifugation for 30 min at 870 *g*. MCMV was constructed as follows. MCMV dm157-luc was constructed based on the MCMV-BAC pSM3fr-MCK-2fl (Jordan *et al.,*^44^ kindly provided by B Adler, Munich, Germany). Deletion of the m157 ORF and insertion of the luc sequence into the m157 locus were performed exactly as described.^45^ Infectious MCMV was reconstituted by the Superfect transfection (Qiagen, Hilden, Germany) procedure. BAC sequences were removed by serial passage in MEF before use of the recombinant virus. Infection efficiency was determined by staining infected cells with CROMA 101, an antibody against viral IE-1 (pp89)^46^ protein before incubating with the secondary DyLight 488 conjugated antibody (#115-485-062, Dianova, Hamburg, Germany, 1 : 300). Staining was performed as described below.

### Plaque assay

The whole plate of macrophages infected with VACV including supernatant was frozen at −80 °C to detach and lyse the cells. After thawing, 200 *μ*l of a dilution series in DMEM/2% FCS was added in duplicates to BSC-40 cells, which had been seeded 1 × 10^5^ cells/well in 24-well plates the day before. After 2 h of incubation at 37 °C, 800 *μ*l of a 1 : 1 mixture of DMEM/10% FCS and 2% methylcellulose was added and incubated for 16 h at standard culture conditions. Medium was removed and cells fixed and stained with 250 *μ*l crystal violet for 20 min. After washing with water and drying, plaques could be counted.

### Western blotting

Whole cells were extracted with lysis buffer (20 mM Tris/HCl pH 7.4; 150 mM NaCl; 10% Glycerol; 1% Triton X-100 and 1x Protease Inhibitor Mix (Roche, Basel, Switzerland)). Protein concentrations were determined using Bradford assay. Following SDS-PAGE, proteins were blotted onto PVDF membranes, and proteins were detected by western blotting. Antibodies used were specific for: Bcl-X_L_ (#54H6, Cell Signaling, Leiden, The Netherlands), Mcl-1 (#600401394, Rockland, Limerick, PA, USA; #1239-1, Epitomics, San Francisco, CA, USA or #559027, BD), VACV F1L (kindly provided by Dr. Antonio Postigo, London), *α*-Tubulin (#T9026, Sigma, Munich, Germany), *β*-Actin (#A5441, Sigma) and GAPDH (#MAB374, Millipore). Signals were detected using horseradish peroxidase-conjugated secondary antibodies (anti-mouse, Dianova, anti-rabbit Sigma IgG) and enhanced chemiluminescence substrate (GE Healthcare, Munich, Germany or Thermo Scientific). Signals were quantified using Intas Lab Image 1D software (Kapelan Bio-Imaging, Leipzig, Germany).

### Mcl-1-specific RNAi and inhibitor

HeLa cells were seeded the day before transfection. siRNA (20 nM final concentration) was mixed with Lipofectamin RNAiMAX (Thermo Scientific) at 1:0.83 v/v in serum-free medium (Optimem, Thermo Scientific), incubated for 20 min at RT and added to the cells. siRNA specific for Mcl-1 (GGCAGTCGCTGGAGATTAT) or scrambled siRNA (termed siCtrl; negative control low GC, #12935-200 Thermo Scientific) was used. Alternatively, the specific Mcl-1 inhibitor A-1210477 (#S7790, Selleck Chemicals, Munich, Germany) was added to the cells (10 μM) after infection with MVA.

### Transient transfection of MEFs

MEF cells were seeded the day before transient transfection. pEGFP-F1L or empty vector (pEGFP-N1, 3.3 *μ*g each) was mixed with FuGene HD (Promega, Mannheim, Germany) (1 *μ*g DNA, 2.5 *μ*l FuGene) in serum-free medium (Optimem, Thermo Scientific), incubated for 15 min at RT and added to the cells.

### Apoptosis and cell death determination by active caspase-3 staining, propidium iodide staining or live/dead cell staining

For cell death assays, floating cells and trypsinized attached cells were combined and washed in PBS. For determination of apoptosis, cells were fixed in 3.7% paraformaldehyde for 20 min at room temperature and incubated in the presence of monoclonal anti-active caspase-3 antibody (#559565, BD Pharmingen, 1:500), before incubating with the secondary Alexa Fluor 647-conjugated antibody (#711-605-152, Dianova, 1:300). Before fixation, a small portion of cells was removed for propidium iodide (PI) staining. In some experiments in macrophages the LIVE/DEAD Fixable Far Red Dead Cell Stain Kit (#L10120, Thermo Scientific) was used. Flow cytometry was performed using a FACS Calibur (Becton Dickinson). In some experiments, the Bcl-2/Bcl-X_L_ inhibitor ABT-737 (#S1002, Selleck Chemicals) was added.

### RNA extraction and quantitative real-time PCR analysis

Total RNA was isolated from either uninfected MEF wt and HeLa cells or the same cells infected with MVA for 24 h using High Pure RNA Isolation Kit (#11828665001, Roche). In all, 1 μg of RNA was reverse transcribed into cDNA using Transcriptor First Strand cDNA Synthesis Kit (#04379012001, Roche). Relative mRNA expression was analysed by quantitative RT-PCR using Light Cycler Taqman Master Kit (#04535286001, Roche) and the Universal Probe Library System (Roche). The following primers and probes were used: hMcl-1: forward aagccaatgggcaggtct, reverse tgtccagtttccgaagcat, probe #4; hHPRT: tgaccttgatttattttgcatacc, cgagcaagacgttcagtcct, probe #73; mMcl-1: cgtgttatgctcccagttcc, aaaatggccagtgaagagca, probe #69; mHPRT: ggagcggtagcacctcct, cctggttcatcatcgctaatc, probe #69. Relative expression of Mcl-1 was normalized to HPRT reference gene expression.

## Abbreviations

MVA: modified vaccinia virus Ankara
VACV: vaccinia virus
MCMV: murine cytomegalovirus
MEF: mouse embryonic fibroblast
Wt: wild type
IFNAR: type I interferon receptor
MOI: multiplicity of Infection
