# Supplementary material for: Anti-apoptotic Bcl-XL but not Mcl-1 contributes to protection against virus-induced apoptosis
Source: Cell Death Dis. 2016 Aug 18;7(8):e2340–. doi: 10.1038/cddis.2016.242 (PMC5108327; doi:10.1038/cddis.2016.242)
Supplement: Supplementary Figures [file cddis2016242x1.pdf]

Suppl. Fig. S1

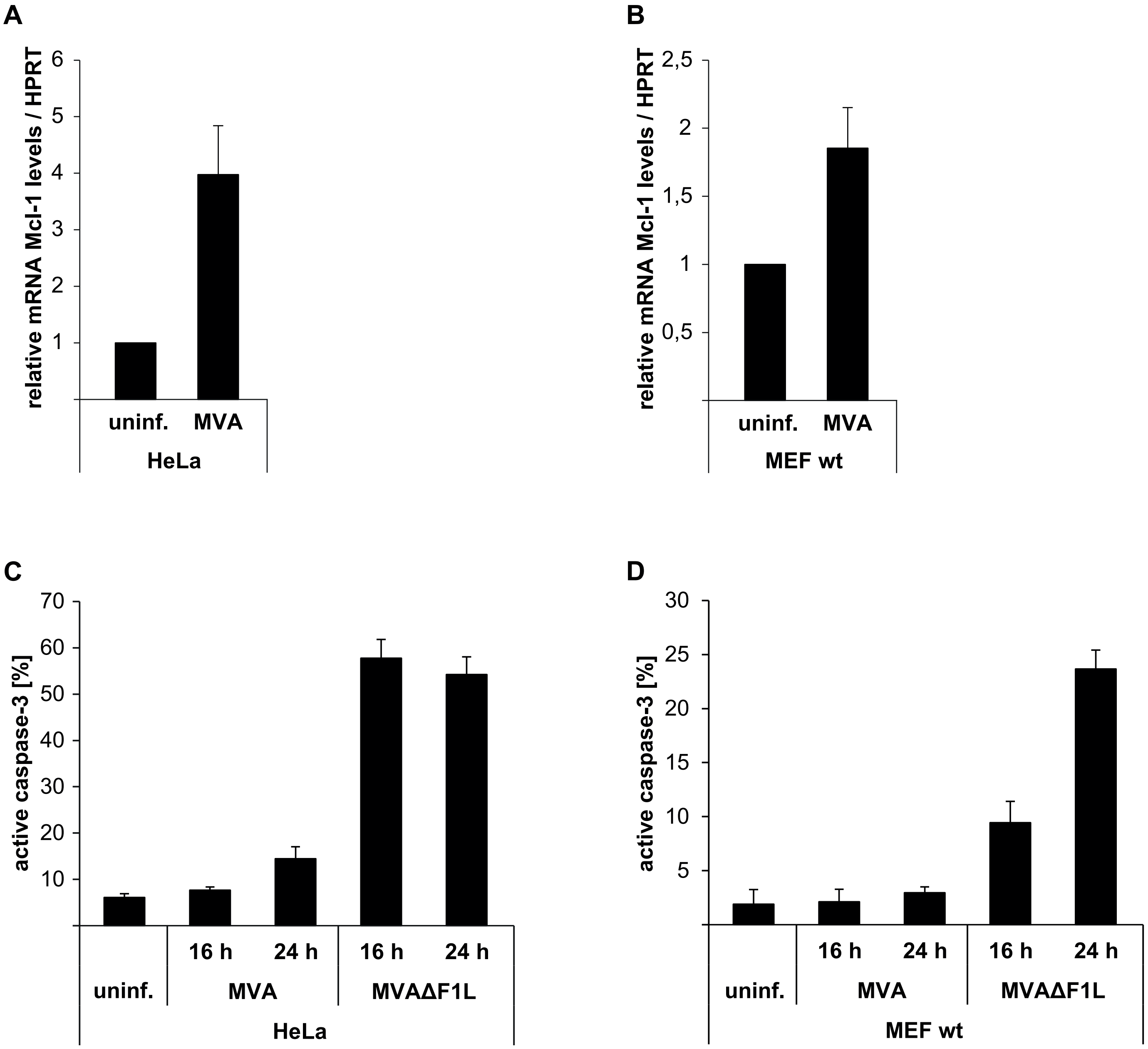

Figure S1

(**A, B**) MVA infection leads to an increase in Mcl-1 mRNA levels in HeLa and MEF cells. Relative Mcl-1 mRNA levels (normalized to HPRT) were measured by qRT-PCR in HeLa (**A**) or MEF wt cells (**B**) after 24 h of infection with MVA. mRNA levels for uninfected cells are set to 1. Data show means/SEM of 3 independent experiments.

(**C, D**) Infection with MVAΔF1L but not MVA leads to apoptosis in HeLa and MEF cells. HeLa (**C**) or MEF wt cells (**D**) were infected with MVA or MVAΔF1L (MOI = 10) for 16 h or 24 h. Apoptosis was measured as the percentage of cells positive for active caspase-3 by flow cytometry. Data show means/SEM of 3 independent experiments.

Suppl. Fig. S2

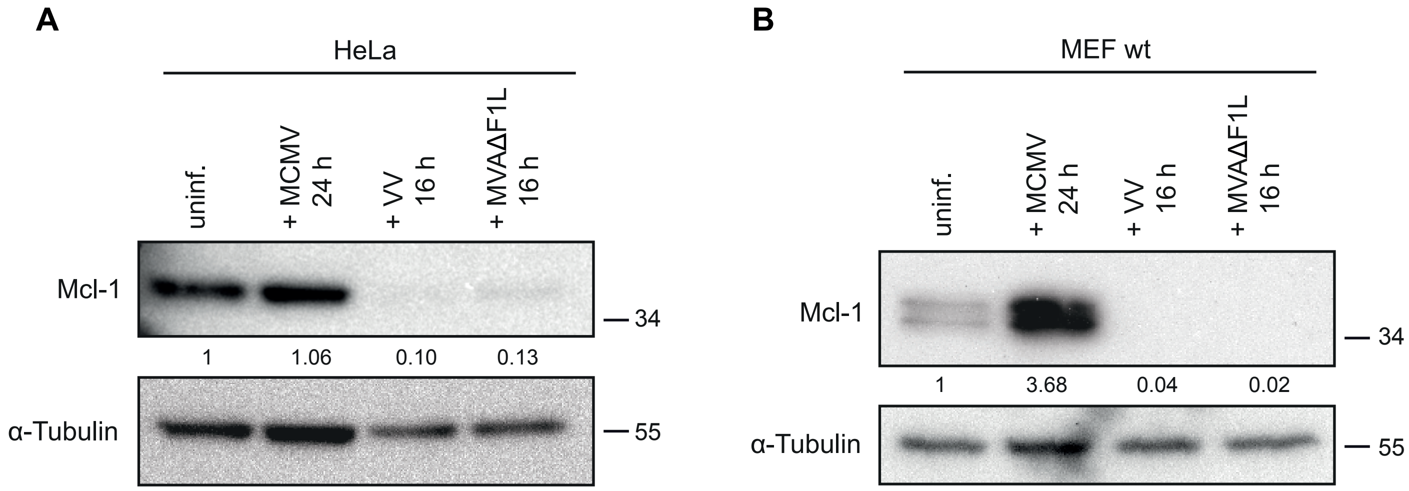

Figure S2

VACV and MVAΔF1L infection leads to a reduction, MCMV infection to no change or an enhanced expression of Mcl-1 in HeLa and MEF cells.
HeLa (**A**) or MEF wt cells (**B**) were infected with MCMV, VACV or MVAΔF1L (all MOI = 10) for the times indicated. Protein levels were determined by Western blotting. Signals were quantified by densitometry and changes in Mcl-1/α-Tubulin ratios normalized to uninfected control are shown below the blots. Data are representative of 3 independent experiments.

Suppl. Fig. S3

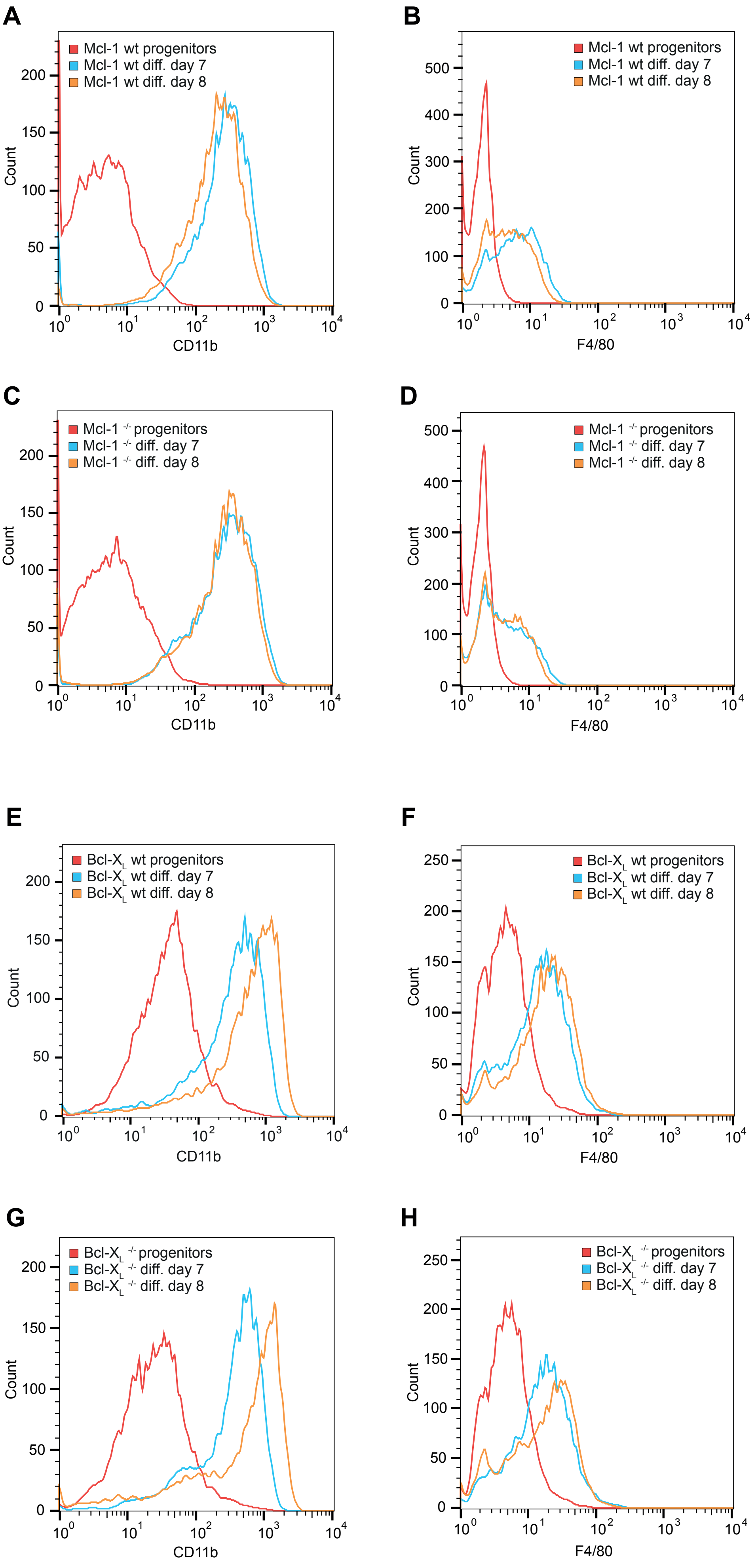

Figure S3

No differences in expression of the myeloid marker CD11b (**A, C, E, G**) and the macrophage-specific marker F4/80 (**B, D, F, H**) can be seen between Mcl-1$^{-/-}$ (**A-D**), Bcl-$X_L^{-/-}$ (**E-H**) and the respective wt cells during macrophage differentiation. Flow cytometry analysis of expression of CD11b (**A, C, E, G**) and F4/80 (**B, D, F, H**) was performed on progenitor cells and differentiated macrophages.
Data are representative of at least 3 independent experiments.

Suppl. Fig. S4

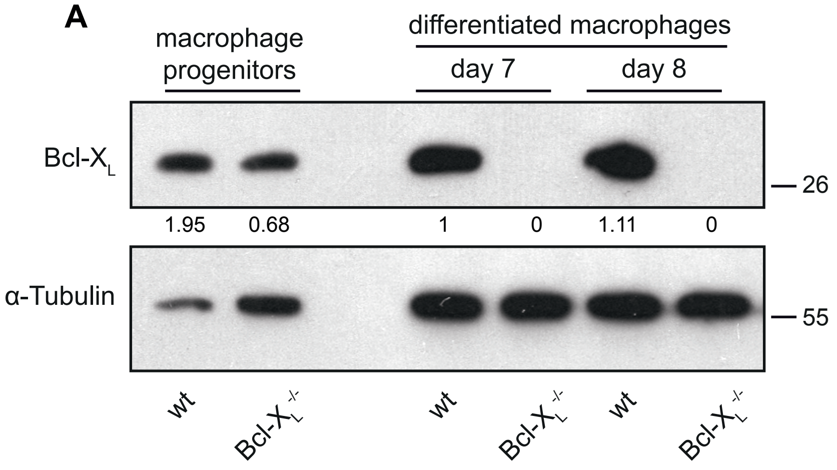

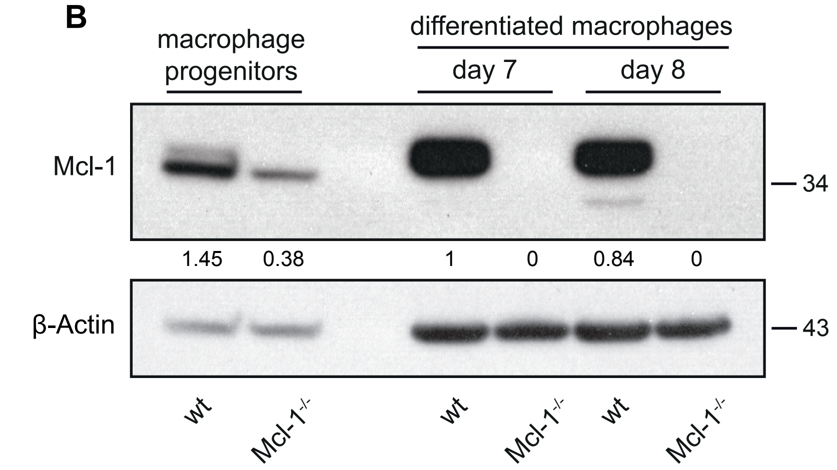

Figure S4

Western blot showing the expression of Bcl-X$_L$ and Mcl-1 in progenitors and differentiated macrophages.

Macrophage progenitors of the genetic background LysM-Cre/Bcl-X$_L^{flox/flox}$ (**A**) or LysM-Cre/Mcl-1$^{flox/flox}$ (**B**) were differentiated for 7 and 8 days alongside the corresponding wt lines and protein levels were assessed by Western blotting. Signals were quantified by densitometry and changes in Bcl-X$_L$/α-Tubulin or Mcl-1/β-Actin ratios normalized to wt control at day 7 of differentiation are shown below the blots. Data are representative of at least 4 independent experiments.

Suppl. Fig. S5

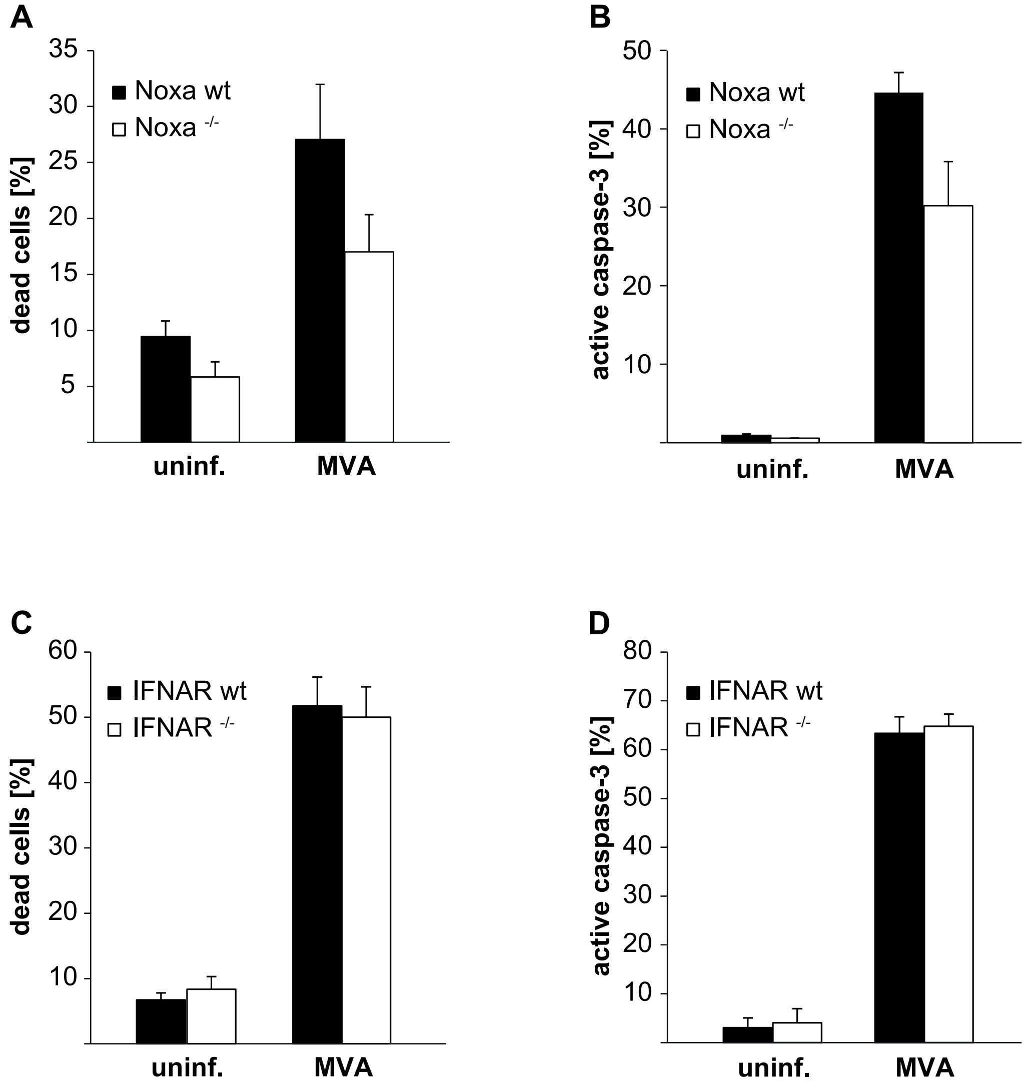

Figure S5

Noxa deficient macrophages are partly protected against MVA-infection (**A, B**) whereas IFNAR$^{-/-}$ macrophages are not (**C, D**).

On day 7 of differentiation Noxa$^{-/-}$ (**A, B**) or IFNAR$^{-/-}$ macrophages (**C, D**), together with the corresponding wt cells were infected with MVA (MOI = 10) for 16 h. Cell death was measured as the percentage of cells positive for the Far Red dye of the Dead Cell Stain Kit (**A, C**) and apoptosis induction as the percentage of cells positive for active caspase-3 (**B, D**) by flow cytometry. Data show means/SEM of 4 (A) or 5 (B, C, D) independent experiments.

Suppl. Fig. S6

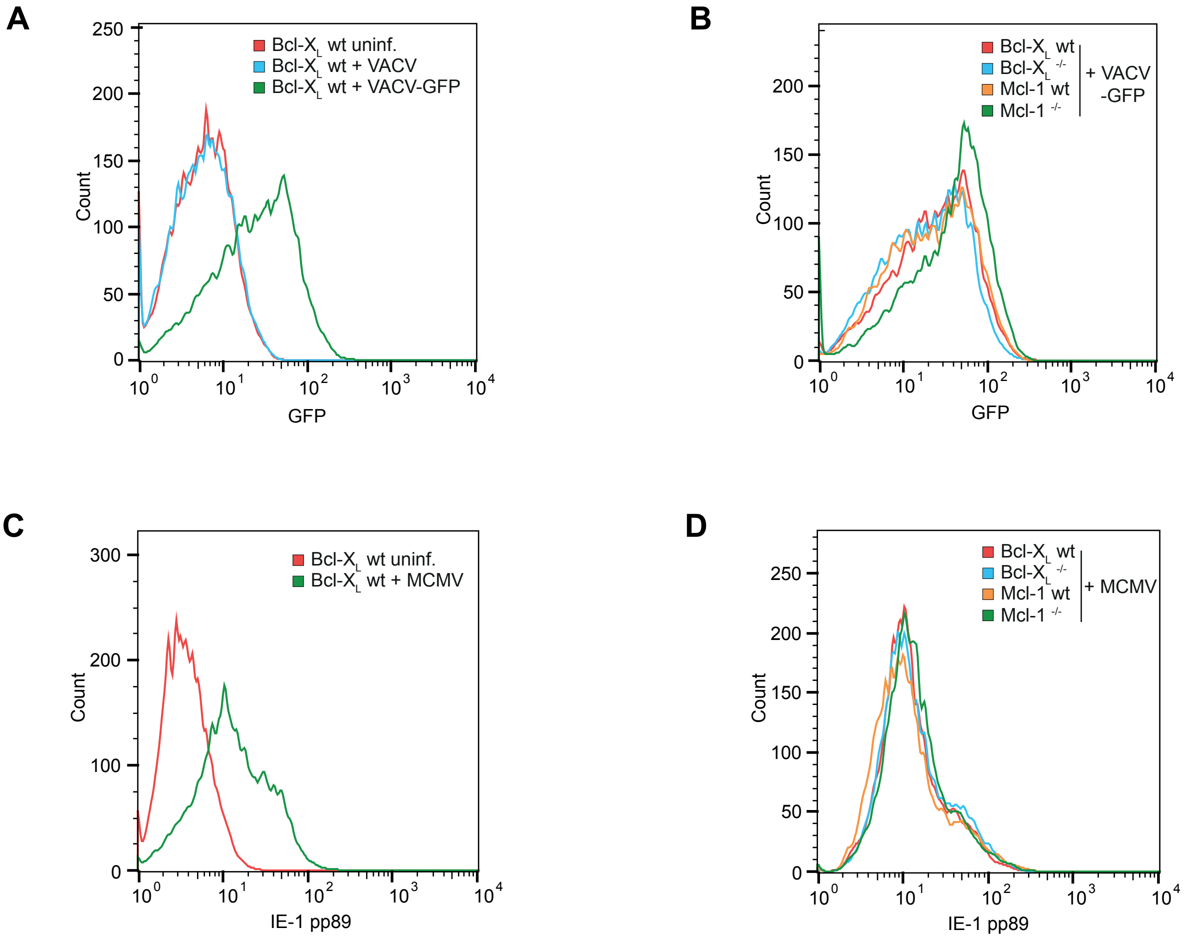

Figure S6

The efficiency of VACV or MCMV infection is comparable in all genotypes in macrophages. On day 7 of differentiation macrophages wt or Bcl-$X_L^{-/-}$ or Mcl-$1^{-/-}$ were infected with VACV (**A**) or VACV-GFP (MOI = 10) for 5 h (**A, B**) or MCMV (MOI = 10) for 10 h (**C, D**). The infection rate was determined by flow cytometry analysis of the expression of viral GFP (**A, B**) or expression of the viral protein IE-1 (pp89) (**C, D**). Data are representative of 3 independent experiments.

Suppl. Fig. S7

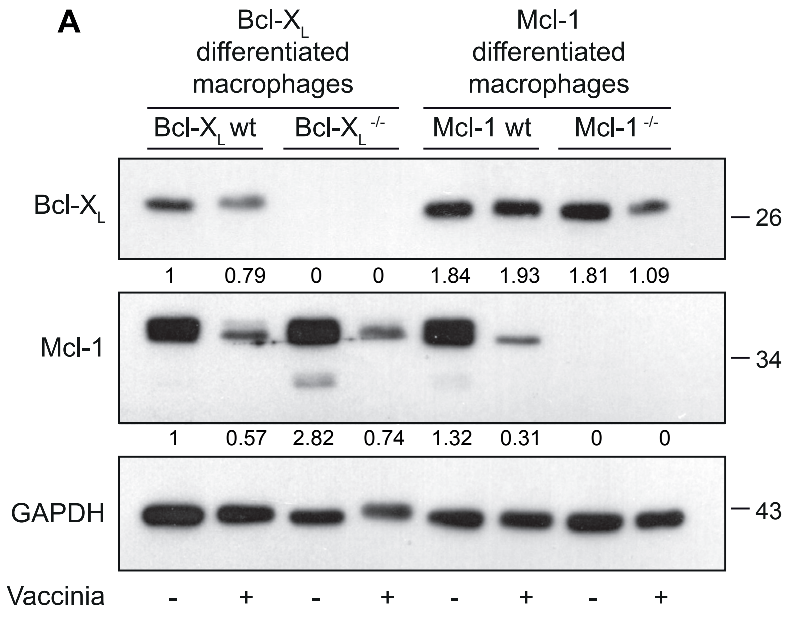

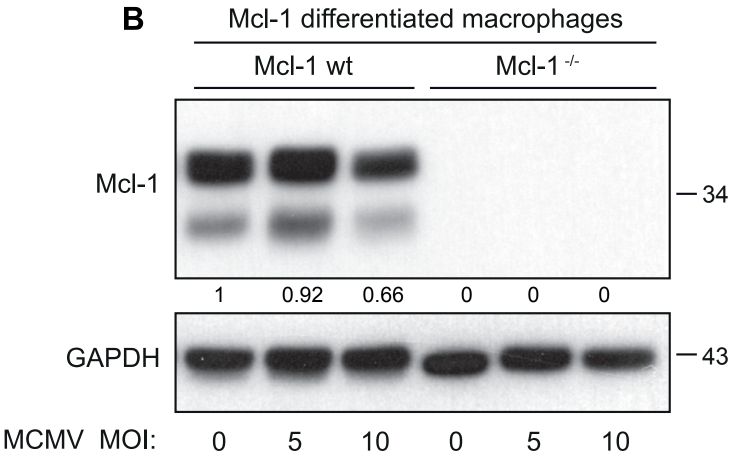

Figure S7

VACV infection leads to a reduction in Mcl-1 levels whereas infection with MCMV does not. (**A**) Macrophages were infected with VACV (MOI = 10) for 16 h. Protein levels were assessed by Western blotting. Signals were quantified by densitometry and changes in Bcl-X$_L$/GAPDH and Mcl-1/GAPDH ratios normalized to uninfected wt control are shown below the blots. Data are representative of at least 3 independent experiments.
(**B**) Macrophages were infected with MCMV (MOI = 5 or 10) for 24 h. Protein levels were assessed by Western blotting. Signals were quantified by densitometry and changes in Mcl-1/GAPDH ratio normalized to uninfected wt control are shown below the blot. Data are representative of at least 3 independent experiments.
